# Effects of single housing on behavior, corticosterone level and body weight in male and female mice

**DOI:** 10.1186/s42826-024-00221-7

**Published:** 2024-09-29

**Authors:** Ilya Smolensky, Kilian Zajac-Bakri, Anne Stephanie Mallien, Peter Gass, Raphael Guzman, Dragos Inta

**Affiliations:** 1 Department of Community Health, University of Fribourg, Chemin du Musée 4, Fribourg, 1700 Switzerland; 2 Department of Biomedicine, University of Basel, Hebelstrasse 20, Basel, 4056 Switzerland; 3 Department of Psychiatry and Psychotherapy, Medical Faculty Mannheim, Central Institute of Mental Health, Heidelberg University, J5, 68159 Mannheim, Germany; 4 Department of Neurosurgery, University Hospital Basel, Spitalstrasse 21/Petersgraben 4, Basel, 4031 Switzerland; 5 Food Research and Innovation Center (FRIC), University of Fribourg, Fribourg, Switzerland

**Keywords:** Single housing, Mice, Behavior, Corticosterone, Body weight

## Abstract

**Background:**

Experimental mice are often single-housed either for an individual analysis (feeding behavior, imaging, calorimetry) or as a stress paradigm (social isolation) in translational biomedical research. Reports of the influence of single housing in rodents are conflicting and may depend on age and duration of isolation. Sex is often not included as a factor. In this study we investigated the effects of 4-week single housing in male and female mice on behavior, body weight, and serum corticosterone levels.

**Results:**

Behavioral tests showed no effect on anhedonia and stress coping, anxiety and motor exploration. Social avoidance occurred in both males and females. Regarding physiological effects, single housing did not induce changes in serum corticosterone levels, but reduced body weight gain.

**Conclusions:**

While some mouse studies of chronic social isolation reported depression-related disturbances, our data suggest that single housing might be not necessarily be too stressful. This is important for animal welfare regulations and experiments in life science research.

## Background

Social isolation is a stressful factor for both humans and many social animals including rodents. Restriction of social contacts during COVID-19 pandemic was considered among the risk factors for mood disorders [1]. In experimental rodents single vs. group housing is an important factor of animal welfare regulations [2], e.g. single housing in early life (post-weaning isolation, maternal separation) represents a classical and widely accepted paradigm of neurodevelopmental disorders like schizophrenia [3].

Animal welfare of in vivo experiments is not only desirable from an ethical point of view, but also contributes to the quality of scientific results [4]. This includes addressing the social needs of the species. Although mice are generally classified as social animals, an adequate form of housing, corresponding to the social structure of wild domestic mice, is not possible under laboratory conditions. There, a habitat is used by a dominant male with several female mice and their offspring. Other males are driven away or killed and subdominants are only rarely tolerated. For this reason, wild males often live alone. Therefore, the required demand of stable and harmonious social groups [5] often turns out to be difficult. In addition to the aggression and potential pain from bite wounds that can occur in groups of males, subdominant animals are sometimes exposed to social defeat over a longer period of time, which can lead to depression-like behavior [4]. Chronic social defeat is also used as an animal model for depression [6]. In addition, male mice from group housing also show tendency for stronger expression of helpless behavior [7]. Sometimes it is therefore even considered sensible to keep male mice individually under certain circumstances [8]. For female mice the situation in quite different as the natural social structure is unbalances. Hence, single housing females can have completely different effects. This poses a problem for the comparability between male and female mice. Stable social housing is supposed to be easier in female groups. After the long-standing prejudice against female mice that the data results fluctuate more has been proven wrong [9] and the demand for the investigation of both sexes in animal experiments is increasingly propagated [10] in order to prevent one-sided research, more and more studies are being carried out with both sexes. Both sexes are often studied in the same housing conditions, although single housing is assumed to be more stressful for female animals than for males. There is evidence of increased anxiety or depressive-like behavior, as well as increased stress hormone release and reduced plasticity markers [11], although there are also studies that show that single housing has no negative effects on stress sensitivity [7] and does not lead to the induction of endocrine and immunological stress reactions [12]. These studies were conducted exclusively in male mice.

We therefore consider it essential to investigate the actual burden of the housing forms for both sexes in this study.

## Methods

### Animals

**Fig. 1:**
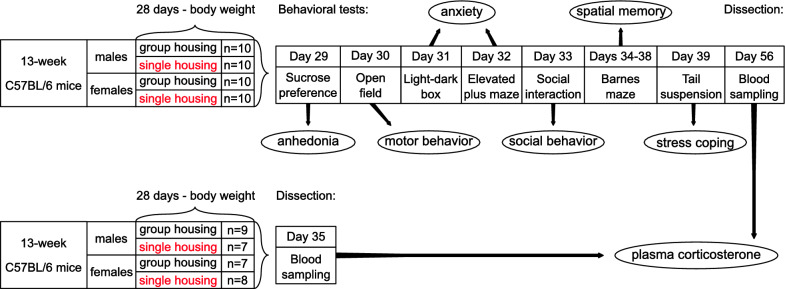
Design of the experiment

All experiments were conducted in accordance with the Swiss animal welfare guidelines under the license №3094, approved by the Cantonal veterinary office of Basel-Stadt. We used male and female C57BL/6 mice (Janvier Labs, Le Genest-Saint-Isle, France) which is the most commonly used mouse line in translational studies. The mice arrived at the animal facility at the age of 6 weeks where they were kept in groups of 4–5 until the start of the experiments. The mice were kept under a 12-hour light cycle (8 a.m.–8 p.m.) with free access to food and water during the whole study. There were four experimental groups (*n* = 17–19, total *N* = 71) used in the study—males vs. females, group-housed vs. single-housed (Fig. 1). Sample sizes were calculated based on the expected effect size of the behavioral outcome parameters according to the previous experiments. Female mice were swapped between litters and male mice from each litter were taken into the isolation group. To minimize the effect of hormonal fluctuation on the females’ outcome their estrous cycles were synchronized, using the Whitten effect—small amounts of bedding from male cages were put into female cages to induce ovulation [13]. All experiments were performed by male researchers, mice were regularly health checked by animal caretakers.

### Single housing

The mice were placed into separate individually ventilated home cages (IVC GM500 for Mice, 38 cm × 20 cm × 17 cm, Tecniplast, Italy) at the age of 13 weeks in order to be physically and olfactorily isolated. The mice stayed in isolation for 4 weeks with bedding and tissue paper as enrichment. The control mice stayed grouped in the similar IVCs (4–5 mice per cage) for the same period of time, all cages were kept in the same rack. One week before and during the 4 weeks of single/group housing mice were weighed and checked twice a week. Behavioral tests were then conducted, while the conditions for the mice remained the same for the rest of the study.

### Behavioral tests

Behavioral studies were performed with a portion of each group (*n* = 10) while another portion (*n* = 7–9) was dissected after 5 weeks of group/single housing for the D35 blood sampling. All behavioral tests were performed from 9 a.m. to 1 p.m. The cages were brought to the experimental room and left there for 30 min for mice to habituate before testing during all experiments. Males and females were tested separately, with breaks to clean the room with ethanol. Tests were performed in a sequence of increasingly stressful impact: sucrose preference, open field, elevated plus maze, light-dark box, social interaction test, Barnes maze, tail suspension test [7]. To decrease stress mice were transferred from the homecage to the apparatus in a plastic lid (from pipette tips box) instead of tunnels to avoid bedding in the apparatus (which might disturb tracking). The apparatus was cleaned with ethanol after each mouse. All tests were video recorded for the further behavioral analysis. The video recordings of the tail suspension test were analyzed manually, whereas the other tests were analyzed by ANY-Maze software (Stoelting Europe, Dublin, Ireland).

### Sucrose preference test (SPT)

Each mouse was put into an individual cage with two bottles—one with water and one with a 2% sugar solution. In half of the cages, the bottle on the left contained water and the one the right contained sweet water, whereas the placement in the other half of the cages was reversed to eliminate lateral preference. After 18 h, the bottles were weighed to calculate the percentage of sucrose consumption as a measurement for depression-like anhedonic behavior [14].

### Tail suspension test (TST)

The TST apparatus consisted of a white box (20 cm × 15 cm, h = 30 cm) with a gap in the ceiling and a hook above it. A plastic tube (2 cm, d = 0.5 cm) was placed on the mouse’s tail to prevent climbing and then a piece of tape was attached to the tail, 3 cm from its base. The mouse was hung on the hook by the tape attached to its tail and remained suspended for 5 min [15]. Video recordings were analyzed by a trained researcher, blinded to the experimental groups, with a keyboard timer software to calculate time and number of immobility episodes (when the mouse did not climb or actively try to escape) as a correlate of the coping strategy. Each mouse was rated at least twice to ensure consistency of the assessment. Where the difference between measured immobility time was > 15 s (14 mice out of 40), third round of the analysis was made to exclude one inconsistent trial or to use all three. The average was considered for the further analysis.

### Elevated plus maze (EPM)

The maze consisted of two open (with a 5 mm fence) and two closed (with 20 cm walls) arms, each measuring 35 cm × 5 cm, and a 5 cm × 5 cm center zone. It was elevated 50 cm above the floor and the open arms were illuminated by two white light lamps with a 25–30 lx brightness. The mouse was placed in the open arm, close to the center zone. The tests lasted for 5 min while the top camera was recording. Time, distance, entries into open arms, and latency to enter the closed arms were calculated to estimate the anxiety level [16].

### Light-dark box (LDB)

The apparatus (h = 20 cm) has two chambers. One was light with transparent walls (25 cm × 25 cm, illumination 500 lx) and one is dark with non-transparent walls with a cover (25 cm × 15 cm) and a 5 cm × 5 cm × 5 cm corridor between them. Mice were placed in the center of the light chamber and tested for 5 min. Time, entries, and average visit duration, and latency to first exit from the light chamber were calculated to estimate anxiety level [17].

### Open field test (OFT)

The open field apparatus was a white square (40 cm x 40 cm) box with 30-cm walls. The arena was illuminated with white light (8–10 lx) and recorded with the top camera. The mouse was placed in the center of the arena for 5 min. Total distance, time, entries and distance in the center zone (10 cm x 10 cm) and in the periphery zone (5 cm from the wall), were calculated to analyze motor activity [18].

### Non-reciprocal social interaction test (SIT)

The social interaction test was performed in the tested mouse’s home cage. After 5-minute familiarization with a metal mesh cup in a home cage all mice and the enrichment were transferred into a temporary cage, and an unfamiliar same-sex intruder mouse was placed under the cup in the corner of the empty home cage. Then the first tested mouse was placed back in its home cage to assess its social behavior. The metal cup allowed for sniffing but prevented fighting and direct physical contact. Then both mice were put into their temporary cages and the next mouse was tested in its home cage with a new intruder after 1 min of habituation. The time spent near the intruder mouse within the 5 min of the test was considered a measurement for the contact (social interaction) [19].

### Barnes maze

The maze was a round plate (d = 75 cm) with 16 round (d = 5 cm) holes along the edge, elevated 80 cm above the floor. The escape box (20 cm x 10 cm, h = 10 cm) was placed under the target hole. The maze arena was brightly illuminated with white light (1000 lx) to create an aversive environment. During habituation day 0, each mouse was first placed into the escape box for 5 min, and then it was placed under the black bucket in the center of the arena for 1 min. After removing the bucket, the mouse was allowed to walk around for 5 min (after that it was carefully directed to the target hole) or until finding the escape box within that timeframe, where it stayed for another 3 min. Subsequently, it was again placed under the bucket to explore the arena until escaping into the box (staying there for 3 min again). Then, the mouse was placed in the arena for the third time (three attempts were enough for all mice to learn how to find the escape box). During learning days 1–3 the escape box’s position was changed compare to day 0, and each mouse was tested twice for 5 min (or until escape) with a 5-10-minute break after 1 min in the escape box. On day 4, the escape box was removed, and each mouse spent 5 min exploring the arena. The distance traveled during learning days 1–3 and the time spent in the target quadrant on day 4 were used to estimate spatial learning and memory [20].

### Serum corticosterone ELISA

After the end of the behavioral tests (6 weeks of single/group housing) mice were left for two more weeks and killed after 8 weeks of isolation (*n* = 10, total *N* = 40). In a separate cohort, the same four groups (*n* = 7–9, total *N* = 31) of mice were kept in isolation for 5 weeks before dissection. The mice were killed by cardiac perfusion under ketamine/xylazine anesthesia. Blood samples (∼0.3 mL) were collected by cardiac puncture, kept at room temperature for 30 min and then centrifuged at 4 °C and 2000*g* for 15 min. The serum supernatant was transferred into another tube, kept on dry ice, and then stored at − 80 °C. Corticosterone concentration was measured using the ELISA kit (Enzo Life Sciences, Farmingdale, New York, USA) according to the manufacturer’s protocol.

### Statistics

Behavioral and biochemical results as well as body weight gain during the four weeks of single housing (difference between values on week 4 and week 0) were analyzed by a two-way ANOVA (housing x sex) followed by the Tukey pairwise comparisons of corresponding groups in case of a significant F-test (*p* < 0.05). Body weight dynamics were analyzed by a two-way repeated measures ANOVA (time x housing), applied separately to males and females. Differences were considered significant for *p* < 0.05. Prism 10 (GraphPad Software, Boston, MA, USA) was used to make graphs and perform statistical analysis.

## Results

### Behavior

#### Anxiety

**Fig. 2:**
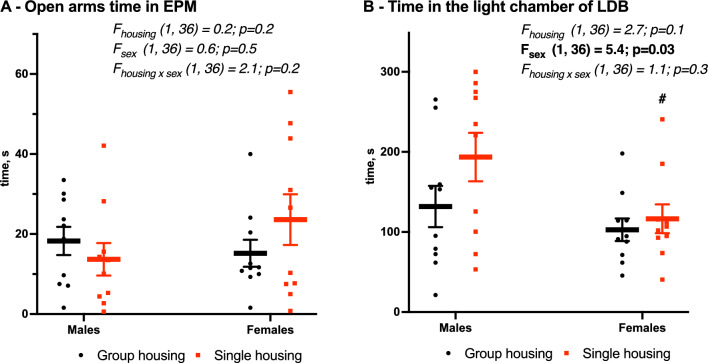
Unchanged anxiety in the elevated plus maze (**A**) and light-dark box (**B**) due to housing condition. Mean (SEM), two-way ANOVA (housing × sex) with Tukey post-hoc test. #Significant sex differences, *p* < 0.05

In the elevated plus maze (Fig. 2A), group-housed and single-housed mice (Table 1) spent a similar amount of time in the open arms (males 18 ± 4 vs. 14 ± 4 s, females 15 ± 3 vs. 24 ± 6 s, F_housing_ (1,36) = 0.2, *p* = 0.2). In the light-dark box (Fig. 2B) the two-way ANOVA showed sex as a factor on time spent in a light chamber (F_sex_ (1,36) = 5.4, *p* = 0.03) which reflects higher anxiety in females than in males. No housing effect was detected.

**Table 1:** Results of behavioral tests

	Males		Females	
	Group-housed	Single-housed	Group-housed	Single-housed
Time in the open arms of EPM (s)	18 ± 4	14 ± 4	15 ± 3	24 ± 6
Entries into the open arms of EPM	3 ± 0.7	3 ± 0.6	3 ± 0.4	4 ± 1.1
Distance travelled in the open arms of EPM (cm)	48 ± 17	27 ± 9	32 ± 11	88 ± 34
Latency to the first enter into the closed arm of EPM (s)	13 ± 3	12 ± 4	15 ± 4	17 ± 4
Time in the light chamber of LDB (s)	132 ± 26	194 ± 30	103 ± 14	116 ± 18^#^
Entries into the light chamber of LDB	9 ± 2	6 ± 1	12 ± 2	10 ± 2
Average visit of light chamber of LDB (s)	28 ± 13	51 ± 20	10 ± 2	13 ± 3^#^
Latency to first exit from the light chamber of LDB (s)	70 ± 30	147 ± 40	35 ± 9	56 ± 22
Total distance travelled on OFT (m)	14 ± 2	12 ± 2	15 ± 2	16 ± 1
Time in the central zone of OFT (s)	4 ± 0.8	5 ± 2	4 ± 1	3 ± 1
Time of thigmotaxis in OFT (s)	131 ± 10	179 ± 17*	156 ± 19	133 ± 11
Entries in the central zone of OFT	5 ± 1	4 ± 1	6 ± 1	6 ± 1
Entries in the thigmotaxis zone of OFT	38 ± 3	31 ± 4	36 ± 3	42 ± 3
Distance in the central zone of OFT (cm)	18 ± 3	18 ± 5	24 ± 5	23 ± 5
Distance of thigmotaxis in OFT (m)	6 ± 1	6 ± 1	6 ± 1	6 ± 1
Latency to enter the thigmotaxis zone of OFT (s)	4 ± 2	7 ± 2	6 ± 1	4 ± 1
Sucrose consumption (%)	64 ± 9	65 ± 10	66 ± 2	82 ± 4
Immobility time in TST (s)	144 ± 18	147 ± 17	96 ± 19	126 ± 17
Immobility episodes in TST	21 ± 2	22 ± 2	18 ± 2	22 ± 2
Time spent in the intruder zone of SIT (s)	197 ± 9	149 ± 10**	183 ± 8	156 ± 10*
Approaches to the intruder in SIT	25 ± 3	37 ± 2	37 ± 3^#^	45 ± 6
Average approach to the intruder mouse in SIT (s)	9 ± 1	4 ± 0.3***	5 ± 0.4^##^	4 ± 0.7
Total distance travelled in Barnes maze (m)	663 ± 115	650 ± 81	488 ± 66	701 ± 119
Time in the target quadrant of Barnes maze on day 4 (s)	147 ± 25	139 ± 16	121 ± 16	117 ± 15

*Significant changes between housing conditions, *p* < 0.05, ** *p* < 0.01, *** *p* < 0.001, # Significant sex differences, *p* < 0.05, ## *p* < 0.01, two-way ANOVA (housing vs. sex) with Tukey post-hoc test. *TST* tail suspension test, *EPM* elevated plus maze, *LDB* light-dark box, *OF* open field, *SIT* social interaction test

### Motor activity

**Fig. 3:**
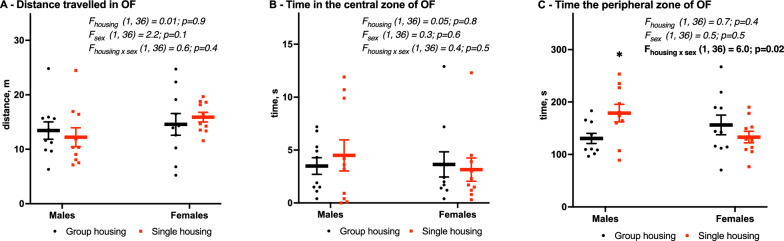
Motor activity in the open field (**A**), time spent in the central (**B**) and the peripheral (**C**) zones. Mean (SEM), two-way ANOVA (housing x sex) with Tukey post-hoc test. *Significant differences between group-housed (black) and single-housed (red) mice, *p* < 0.05

Total distance travelled in the open field (Fig. 3A) was similar across the groups (13 ± 2 vs. 12 ± 2 m in males, 15 ± 2 vs. 16 ± 1 m in females, F_housing_ (1,36) = 0.01, *p* = 0.9), same as the time spent in the central zone (3.5 ± 0.8 s vs. 4.5 ± 1.5 s in males, 3.6 ± 1.1 s vs. 3.2 ± 1.1 s in females, F_housing_ (1,36) = 0.05, *p* = 0.8, Fig. 3B). Time spent in the peripheral zone (thigmotaxis, F_housing x sex_ (1,36) = 6.0, *p* = 0.02, Fig. 3C) was increased in single-housed males (130 ± 10 vs. 179 ± 17, *p* < 0.05) but stayed unaffected in females (156 ± 19 vs. 133 ± 11 s).

### Anhedonia

**Fig. 4:**
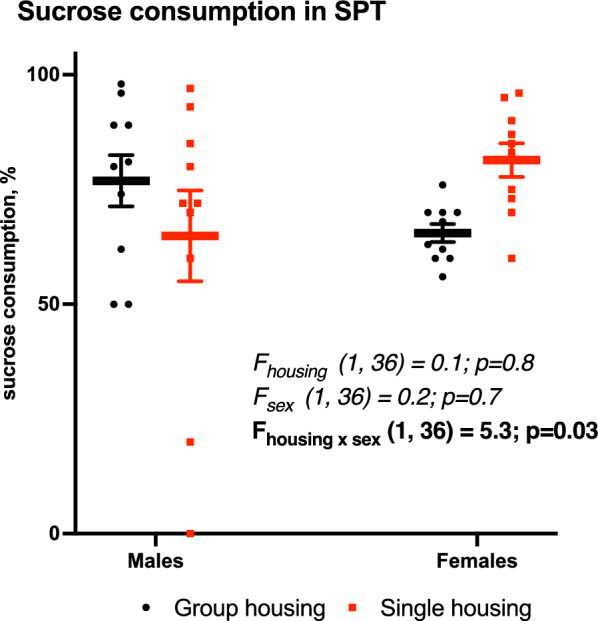
Unchanged anhedonia in sucrose preference test (SPT) due to housing condition. Mean (SEM), two-way ANOVA (housing × sex) with Tukey post-hoc test

In the sucrose preference test, group-housed and single-housed mice consumed comparable rates of sweet solutions (65–82%, F_housing_ (1,36) = 0.1, *p* = 0.8, Fig. 4).

### Stress coping

**Fig. 5:**
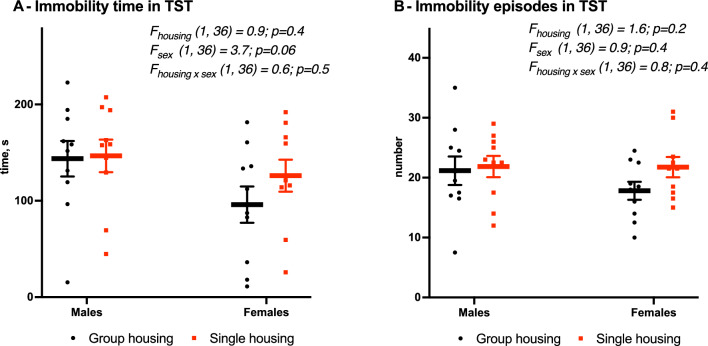
Unchanged stress coping in tail suspension test (TST) due to housing condition. **A **Time of immobility, **B** Episodes of immobility. Mean (SEM), two-way ANOVA (housing × sex)

In the tail suspension test, mice of all groups showed similar duration (F_housing_ (1,36) = 0.9, *p* = 0.4, Fig, 5 A) and number (F_housing_ (1,36) = 1.6, *p* = 0.2, Fig. 5B) of immobility episodes.

### Social interaction

**Fig. 6:**
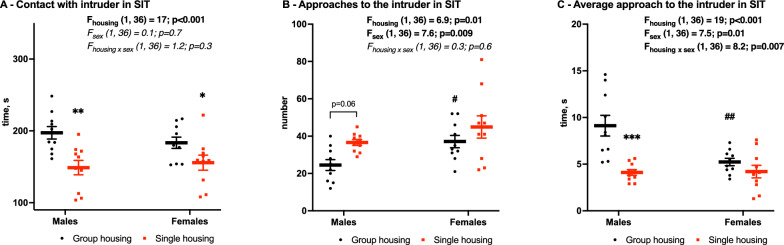
Time of contact with the intruder (**A**), number (**B**), and average duration (**C**) of contacts in social interaction test (SIT). Mean (SEM), two-way ANOVA (housing × sex) with Tukey post-hoc test. *Significant differences between group-housed (black) and single-housed (red) mice, *p* < 0.05, **p < 0.01, #Significant between males and females, *p* < 0.05, ##*p* < 0.01

Social behavior was analyzed using the non-reciprocal social interaction test. Contact (time spent around the mesh cup with intruder mouse) significantly decreased after a 4-week single housing (F_housing_ (1,36) = 17, *p* < 0.001, Fig. 6A) independent from sex: in males from 197 ± 9 to 149 ± 10 s (*p* = 0.003) and females from 183 ± 8 s to 156 ± 10 s (*p* = 0.04). However, the number of approaches (entries to the intruder zone) is increased (F_housing_ (1,36) = 6.9, *p* = 0.01, Fig. 6B). Females showed more approaches than males (F_sex_=1.36, 7.6, *p* = 0.0009). A decreased total time of contact with a potentially increased number of approaches in males resulted in a corresponding decrease in average duration of a single contact episode (F_housing_ (1,36) = 19, *p* < 0.001; F_sex_ (1,36) = 7.5, *p* = 0.01; F_housing×sex_ (1,36) = 8.2, *p* = 0.007) from 9.1 ± 101 s to 4.1 ± 0.3 s (*p* < 0.001, Fig. 6C). Also, contact episodes with an intruder mouse were shorter in group-housed females than in group-housed males (5.2 ± 0.4 s vs. 9.1 ± 1.1 s, *p* = 0.002, Fig. 6C).

### Spatial memory

**Fig. 7:**
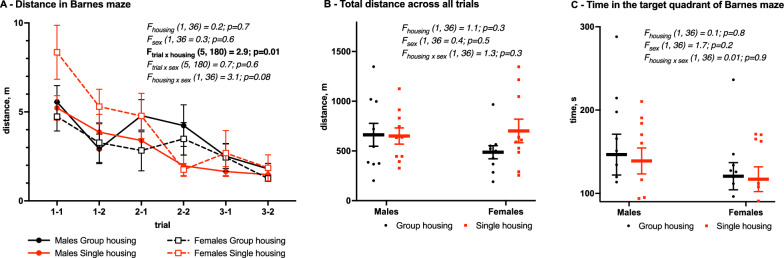
Spatial memory in Barnes maze due to housing condition. Mean (SEM). **A **Distance travelled during each trial (**A**), three-way repeated measurements ANOVA (trial vs. housing vs. sex). **B **Total distance travelled during three days of learning, **C **Time spent in the target quadrant on the day 4. Two-way ANOVA (housing vs. sex)

Spatial memory was analyzed using the Barnes maze (Fig. 7). Three-way repeated measurements ANOVA (housing vs. sex vs. trials) did not show a significant effect of the overall housing factor on the distance to reach the escape box during the three days of learning (F_housing_ (1, 36) = 0.2, *p* = 0.7, Fig. 7A). Significant trial × housing interaction (F_trial×housing_ (5, 180) = 2.9, *p* = 0.01) suggests that single housing induces memory disturbance, however, the analysis of total distance travelled during six trials (F_housing_ (1, 36) = 1.1, *p* = 0.3, Fig. 7B) does not confirm this. Time spent in the target quadrant on day 4 was also not impacted by single housing (F_housing_ (1, 36) = 0.1, *p* = 0.8, Fig. 7C).

#### HPA axis corticosterone

**Fig. 8:**
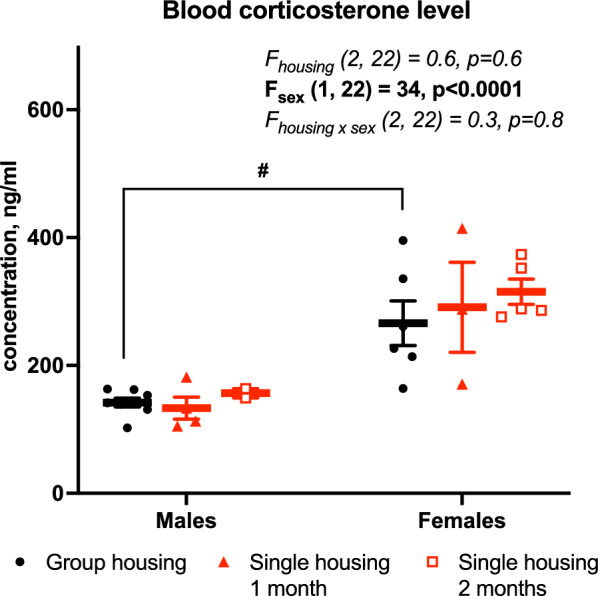
Blood corticosterone level, Mean (SEM). Two-way ANOVA (housing vs. sex) with Tukey post-hoc test, #Significant differences by sex, *p* < 0.05

Corticosterone levels were measured in serum samples after one and two months of single housing (Fig. 8). A two-way ANOVA did not show a significant effect of housing (F_housing_ (2, 22) = 0.6, *p* = 0.6). Meanwhile, significant differences between the sexes were found (F_sex_ (1, 22) = 36, *p* < 0.0001), showing that corticosterone levels were higher in group-housed females than in group-housed males (266 ± 35 pg/ml vs. 142 ± 7 pg/ml, *p* < 0.05).

### Body weight

**Fig. 9:**
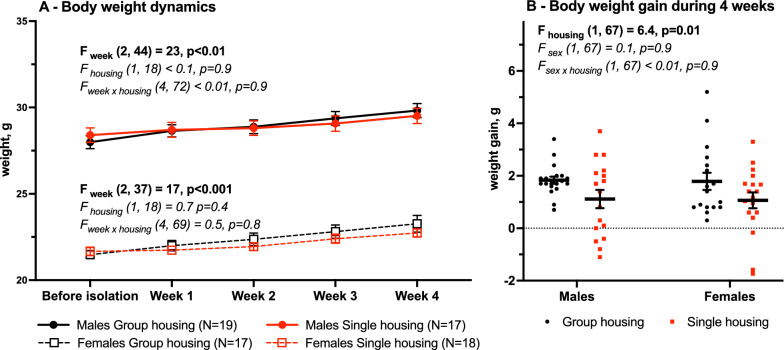
Body weight changes during 4 weeks of group or single housing, Mean (SEM). **A** Dynamics, two-way repeated measurements ANOVA (week vs. housing), **B** Weight difference between week 0 and week 4 of group/single housing, two-way ANOVA (housing vs. sex) with Tukey post-hoc test

Body weight was measured twice a week, one week before the experiment, and during the first 4 weeks of single housing (Fig. 9). The two-way ANOVA (housing vs. week) did not find an effect of the housing factor on the body weight dynamic throughout the 5 weeks (males: F_housing_ (1,18) < 0.1, *p* = 0.9; females: F_housing_ (1,18) = 0.7, *p* = 0.4, Fig. 9A). However, body weight gain over 4 weeks of single housing was lower in group-housed mice F_housing_ (1,67) = 6.4, *p* = 0.01, Fig. 9B).

## Discussion

In this study, we investigated the effect of a 4-week single housing on behavioral and physiological parameters of male and female mice. While some studies reported significant stress-related changes in behavior, body weight, and blood hormones by social isolation (see Table 2), our results show almost no differences between group-housed and single-housed mice, both males and females.

**Table 2:** The results of current study compared to other studies in mice

Study	Strain, sex, length of isolation (weeks of life)	Serum CORT	Body weight	Anxiety	Immobility	Motor activity	Cognitive function	Social behavior
Current study	Male and female C56BL/6J 4 weeks (14–17)	No effect in M and F	No effect in M and F	No effect in EPM and LDB in M and F	No effect in TST in M and F	No effect in OF in M and F	No effect in BM in M and F	Social avoidance in non-reciprocal SIT in M and F
Abramov et al. [30]	Male and female C56BL/6J 4 weeks (14–17)		Decreased in M but not in F	Decreased in M, increased in F in EPM				Aggression in M, increased communication in F in reciprocal SIT
Berry et al. [11]	Male C56BL/6J 3 weeks (?)	Exaggerated stress response		Increased in EPM	Increased in FST			No effect in reciprocal SIT
Guo et al. [25]	Male and female Swiss–Kunming 13 weeks (4–16)		Decreased in M and F	Decreased in EPM (in M) and in LDB (in M and F)	Decreased in FST in M	Increased in OF in M but not in F		
Kumari et al. [43]	Female C56BL/6J 8 weeks (10–17)			Increased in EPM and OFT				
Lander et al. [23]	Male C56BL/6 3 weeks (9–11)		No effect	Increased in OFT			No effect	
Lee et al. [27]	Male C56BL/6 N 4 weeks (10–13)	Increased		Increased in OFT	Increased in FST and TST			
Liu et al. [31]	Male C56BL/6J 10 weeks (9–18)							Social avoidance in non-reciprocal SIT
Liu et al. [22]	Male and female C56BL/6J 8 weeks (8–15)			Increased in MBT in M and F	Increased in TST in M and F, in FST in M		Decreased in MWM, NOR, Y-maze in M and F	
Rivera-Irizarry et al. [21]	Male and female C56BL/6J 6 weeks (10–15)			Increased in LDB (but not in EPM) in M and F				No effect in social preference test in M and F

*M* males, *F* females. *EPM* elevated plus maze, *LDB* light-dark box, *FST* forced swimming test, *TST* tail suspension test, *OF* open field, *SIT* social interaction test, *BM* Barnes maze

Some studies reported that prolonged single housing might increase anxiety in different tests in both sexes [21–23] or only in males [11]. Despite some questions about the predictive validity of EPM and LDB for anxiety measurement [24], our data show no changes in these tests in single-housed males and females consistent with some similar studies [25]. Motor activity in the OFT was also unaffected, although single-housed males spent more time near the wall than group-housed.

Anhedonia measured by a rate of sucrose consumption is often use to estimate depression-like behavior since it mimics a reduced motivation to seek for pleasure in depressed patients [26]. However, none the studies with single-housed mice estimated their anhedonia in SPT before. Immobility in TST and FST has been reported to increase in some studies [11, 22, 27] while others found it to be decreased [25]. However, current consensus is that immobility in these tests reflects rather stress coping strategy (active escape vs. passive floating/hanging until being “saved”) than despair and might therefore reflect adaptive response [28, 29]. Our results do not show any effect of single housing on the immobility time, on contrary to several previous studies mentioned above.

Single housing-induced cognitive deficits in mice were reported only in one study and it included both sexes [22]. Single-housed males and females showed a disturbed spatial memory in the Morris water maze and Y-maze, as well as declined object memory in the Novel object recognition test. In our experiment, a 4-week single housing did not disturb spatial memory and learning in the Barnes maze—both the distance travelled to find an escape box during three days of training and the time spent in a target quadrant on acquisition day (Day 4) stayed unaffected.

The only significant effect of single housing, which we have found in our study, was social avoidance. Both male and female mice spent significantly less time exploring unfamiliar same-sex mouse confined in a mesh cup. Meanwhile, the structure of social behavior also changed in males—they seemed to make more approaches, however, each of them was shorter on average. Social disturbances were reported in several studies of single housing and they varied across the studies, depending dramatically on the applied social test. We used non-reciprocal SIT, where the intruder mouse is confined by a mesh cup to prevent direct contact and fights, while in reciprocal SIT mice are allowed to freely interact in a cage. In reciprocal SIT single housing might increase aggression in males and non-aggressive communication in females [30], while in non-reciprocal SIT it usually has no effect on social behavior [11, 21] or induces social avoidance similar to our results [31].Therefore, results of social tests across the studies with single housing are of a wide variability depending on sex and reciprocal or non-reciprocal variant of test (See Table 2). Single housing-induced social avoidance, which we found in our experiments, has previously not been reported in adult females while only one study also used non-reciprocal SIT [21]. Nevertheless, given the absence of any other stress-related impairments in this study, we assume that social disturbances might be associated with the isolation itself rather than with social stress. However, we did not find any studies where single housing affected only social behavior without influencing anxiety or depressive-like behavior as well. Moreover, two works with anxiety, anhedonia, and immobility in TST or FST in isolated mice [11, 21] did not find aggression or social avoidance.

We measured basal corticosterone level at two time points—after one and two months of the experiment, and it did not show any effect of single housing on the HPA axis activity in males (stayed around 150 ng/ml) and females (stayed around 250–300 ng/ml). One study also found no effect on basal corticosterone level, but its stress-induce elevation was exaggerated in isolated males (from ~ 160 ng/ml to ~ 220 ng/ml) [11]. However, another study reported increased basal corticosterone level (from ~ 100 ng/ml to ~ 275 ng/ml) [27] while no data are available in single-housed females. A big review of animal stress models reported that 91% of 120 included studies found an elevated basal corticosterone level, which makes it a reliable marker of chronic HPA axis hyperactivity [32]. Meanwhile, our results showed no signs of stress-related hormonal response in single-housed mice.

Changes in the appetite and body weight is an often consequence of stress as well as one of the major depressive disorder symptoms including both body weight loss (melancholic depression) and body weight gain (atypical/immunometabolic depression) [33]. Rodent studies often report stress-induced body weight changes which also might be both increased and decreased. Single housing of both sexes resulted in body weight loss only in males [30] or in both males and females [25]. One study in males reported no effect of isolation in males [23], which we observed in both sexes. However body weight gain over the 4 weeks of isolation was smaller than in group-housed males and females which might reflect some mild effect on the mice wellbeing.

It is worth mentioning a substantial difference in the effects of single housing in mice versus rats, suggesting important species-specific mechanisms. In a vast majority of studies with adult rats single housing resulted in behavioral disturbances [34–37]. Studies conducted in males and females reported anxiety and anhedonia as well as decreased (not increased) corticosterone in both sexes [34, 38]. Long-term single housing might be more harmful for rats than for mice due to their ecological differences in nature. Rats are highly social and live in mixed-sex groups of 10–15 animals with rare fights [39] whereas male mice live solitarily and show high territorial-related aggression towards other males [40]. Female mice usually live together in the territory of one male, but they do not have tight social bonds. These significant differences between mice and rats should be considered in social tests and social stress paradigms including rodent models of affective disorders [41, 42].

Our study shows that a 4-week single housing of adult male and female mice does not have severe effects on their behavior and blood corticosterone level while reduced body weight gain over time.

As discussed above, some other similar studies also did not find any effect of single housing on anxiety and stress coping [25], body weight and memory [23]. Others, though, reported increased anxiety [11, 23, 27, 43], anhedonia and immobility [11, 22, 27], memory decline [22], body weight loss [25, 30], and HPA axis activation [11, 27]. The only significant effect by single housing in our study was a social avoidance in both males and females, while other studies reported the whole spectrum of social behavior from aggression [30] to social avoidance [31] as well as increased [30] or unchanged [11, 21] social interaction.

## Conclusions

In sum, our results do not confirm many other reports of behavioral and physiological disturbances indicating a high variability of experimental outcomes. It might mean that single housing for 4 weeks is less harmful than it is currently considered by the animal welfare regulations. This contrasts with reliable long-term alterations triggered by the same isolation paradigm, when exposure starts at earlier, post-weaning stages. It suggests an increased resilience to prolonged single housing after adolescence. Future studies may unravel the neurobiological correlates of the maturation of circuitry, which underlie resilience to isolation [44], as well as the differential responsiveness in different species, such as in mice versus rats. In a larger sense, our results suggest that single housing, as necessary in various investigational paradigms (as for using special devices/cages etc.), may not represent a significant bias, affecting the wellbeing of the analyzed mice and the outcome of such experiments. Therefore, the current data are also of importance for a reevaluation of regulation in animal experimentation. Current regulations regarding stress/housing often do not distinguish between developmental (post-weaning, adolescent) and adult stages or rodent species (mice or rats), considering, for example, chronic single housing during adulthood as a severe stressor, due to its long duration.

A limitation of both our study and most of publications is that they were done in C57BL/6 mice. It is the most commonly used mouse line in many fields of translational biomedicine including neurobiology, however to which extend these conclusions apply to other lines (such as BALB/c, ICR, Swiss albino) remains to be investigated.

## Data Availability

All data from the study is available here https://osf.io/zg39m/.

## Abbreviations

EPM: Elevated plus maze
ELISA: Enzyme–linked immunosorbent assay
IVC: Individually ventilated cage
LDB: Light–dark box
OF: Open field
RM ANOVA: Repeated measures analysis of variance
SIT: Social interaction test
TST: Tail suspension test
