# Correction: Effects of single housing on behavior, corticosterone level and body weight in male and female mice

**DOI:** 10.1186/s42826-024-00223-5

**Published:** 2024-10-29

**Authors:** Ilya Smolensky, Kilian Zajac-Bakri, Anne Stephanie Mallien, Peter Gass, Raphael Guzman, Dragos Inta

**Affiliations:** 1 Department of Community Health, University of Fribourg, Chemin du Musée 4, Fribourg, 1700 Switzerland; 2 Department of Biomedicine, University of Basel, Hebelstrasse 20, Basel, 4056 Switzerland; 3 Department of Psychiatry and Psychotherapy, Medical Faculty Mannheim, Central Institute of Mental Health, Heidelberg University, J5, 68159 Mannheim, Germany; 4 Department of Neurosurgery, University Hospital Basel, Spitalstrasse 21/Petersgraben 4, Basel, 4031 Switzerland; 5 Food Research and Innovation Center (FRIC), University of Fribourg, Fribourg, Switzerland

**Correction to: Lab Anim Res 40, 35 (2024)**

10.1186/s42826-024-00221-7

Following publication of the original article [1], the authors reported that “C56Bl/6J” in Methods, and “C56BL/6 mice” in Conclusion and Fig. 1 should be corrected to “C57BL/6 mice”.

The originally published wrong sentence in Animals section of Methods was:

We used male and female C56Bl/6J mice (Janvier Labs, Le Genest-Saint-Isle, France) which is the most commonly used mouse line in translational studies.

The correct sentence in Animals section of Methods should read:

We used male and female C57BL/6 mice (Janvier Labs, Le Genest-Saint-Isle, France) which is the most commonly used mouse line in translational studies.

The originally published wrong sentence in Conclusion was:

A limitation of both our study and most of publications is that they were done in C56BL/6 mice. It is the most commonly used mouse line in many fields of translational biomedicine including neurobiology, however to which extend these conclusions apply to other lines (such as BALB/c, ICR, Swiss albino) remains to be investigated.

The correct sentence in Conclusion should read:

A limitation of both our study and most of publications is that they were done in C57BL/6 mice. It is the most commonly used mouse line in many fields of translational biomedicine including neurobiology, however to which extend these conclusions apply to other lines (such as BALB/c, ICR, Swiss albino) remains to be investigated.

The originally published Fig. 1 was:

**Fig. 1:**
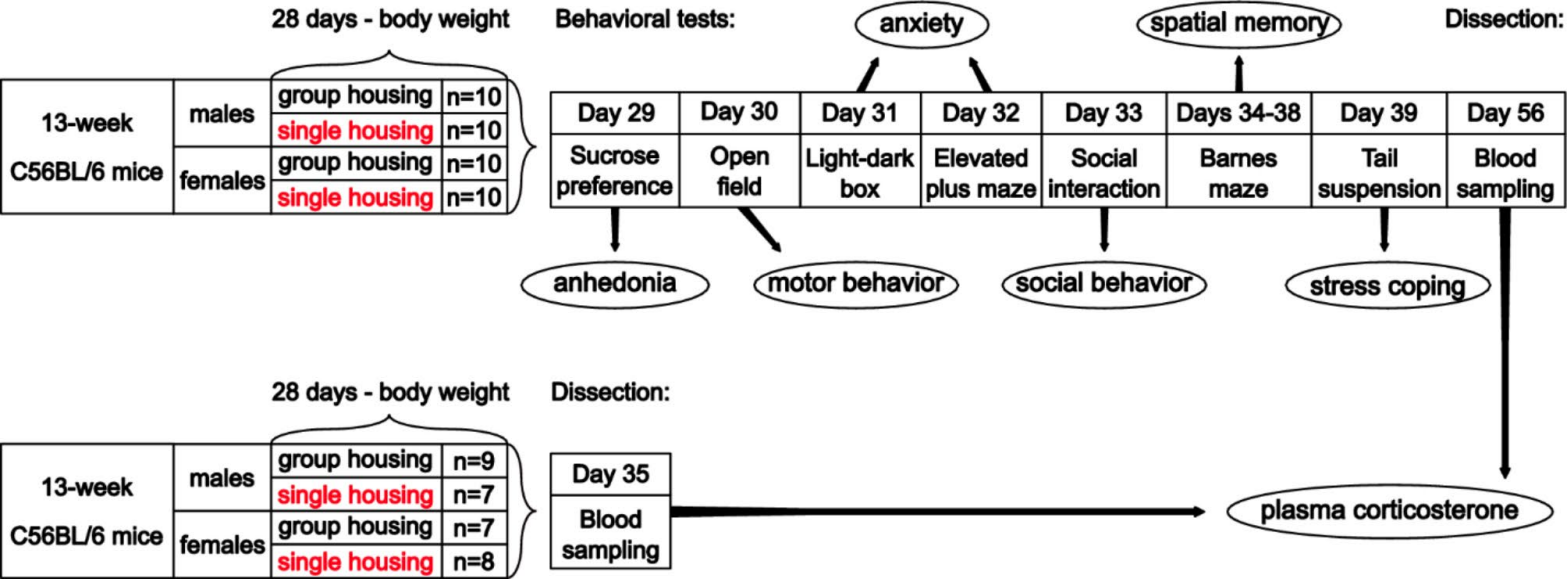
Design of the experiment

The correct Fig. 1 should read:

**Fig. 1:**
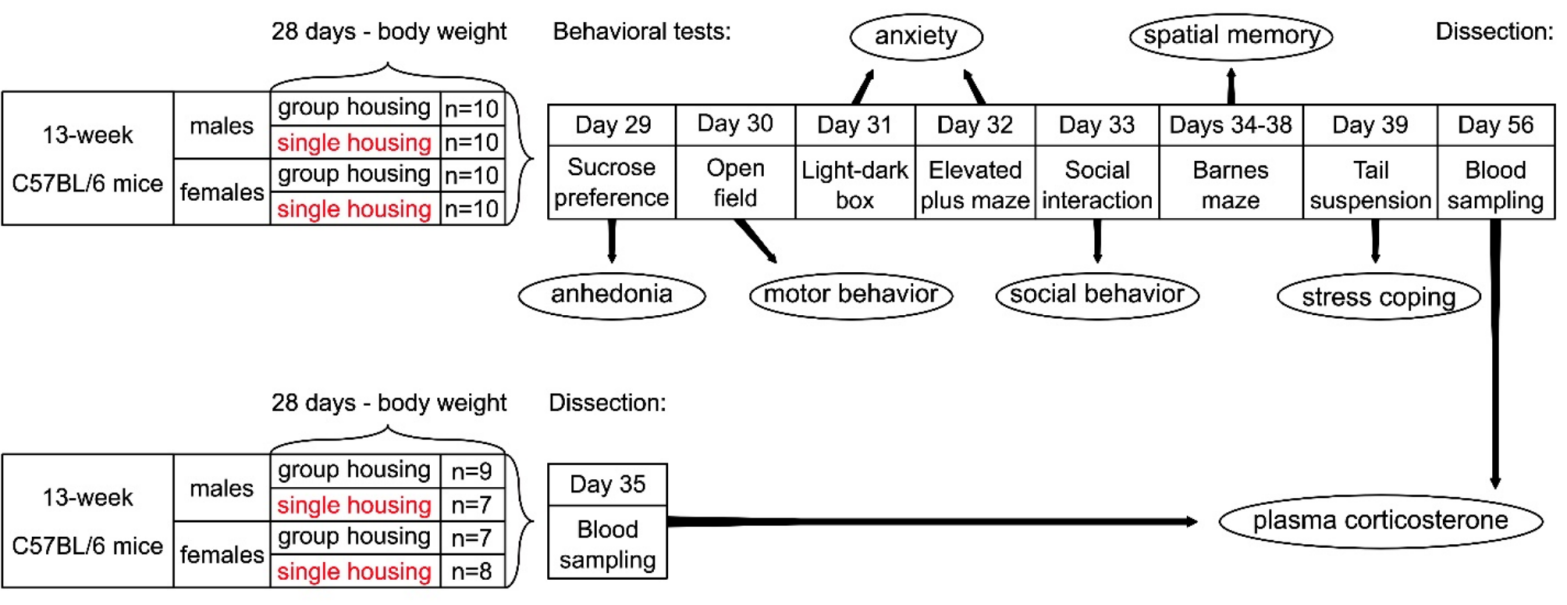
Design of the experiment

The original article [1] has been updated.
